# Supplementary material for: Anti-Cancer Effects of Artesunate in Human 3D Tumor Models of Different Complexity
Source: Int J Mol Sci. 2023 Apr 25;24(9):7844. doi: 10.3390/ijms24097844 (PMC10178545; doi:10.3390/ijms24097844)
Supplement: Supplementary file 1 [file ijms-24-07844-s001.zip › ijms-2360088-supplementary-Table S3.pdf]

Table S3. Statistical correlation between Study cohort parameters and Efficacy of Artesunate.

Cohort Parameter		Effect	
		< 13.87	> 13.87
Age	≤ 58 Jahre	9	11
	> 58 Jahre	13	6

Cohort Parameter		Effect	
		< 13.87	> 13.87
Gender	Female	15	10
	Male	7	7

Cohort Parameter		Effect	
		< 13.87	> 13.87
Localisation	Breast Cancer	9	5
	Non-Breast Cancer	13	12

Cohort Parameter		Effect	
		< 13.87	> 13.87
Tumor status	Primary	8	4
	Progressed	14	13

p-value, calculated with 2-tailed Fisher's exact test
**p = 0.200**

p-value, calculated with 2-tailed Fisher's exact test
**p = 0.738**

p-value, calculated with 2-tailed Fisher's exact test
**p = 0.518**

p-value, calculated with 2-tailed Fisher's exact test
**p = 1.000**
