# Supplementary material for: Anti-Cancer Effects of Artesunate in Human 3D Tumor Models of Different Complexity
Source: Int J Mol Sci. 2023 Apr 25;24(9):7844. doi: 10.3390/ijms24097844 (PMC10178545; doi:10.3390/ijms24097844)
Supplement: Supplementary file 1 [file ijms-24-07844-s001.zip › ijms-2360088-supplementary-Tables S1 and S2.pdf]

Table S1. Overview of Artesunate in Clinical Anti-Neoplasia Trials.

Author/ Year	*Chemo-therapy*	*Disease*	*Study Phase*	*Study size*	*Dosing*	*Duration*	*Form of application*	*Peak plasma concentration*	*Anti-tumor effect*	*Toxicities*	*Reference*
Jansen, 2011	None	Cervical Cancer	Phase 1	10	100 mg<br><br>200 mg	28 days	Oral	Not measured	Down-regulation of p53, EGFR, Ki67 and CD31<br>Up-regulation of CD71<br>No changes in tumor size<br>First remission in all patients<br><br>Median survival time of 12 months	Flu-like syndrome (1/2)<br><br>Headache (1/2)<br><br>Abdominal pain (1/2)	Jansen FH, Adoubi I, J C KC, DE Cnodder T, Jansen N, Tschulakow A, Efferth T. First study of oral Artenimol-R in advanced cervical cancer: clinical benefit, tolerability and tumor markers. Anticancer Res. 2011 Dec;31(12):4417-22. PMID: 22199309.
Krishna, 2014	None	Colorectal Cancer	Phase 1	23	200 mg	14 days	oral	Not measured	Apoptosis of >7% of tumor cells in 67% in intervention vs. 55% in control group<br>2 year survival of 91% in intervention vs. 53% control group<br>Decreased expression of Ki67	Neutropenia, anemia, nausea	Krishna S, Ganapathi S, Ster IC, Saeed ME, Cowan M, Finlayson C, Kovacsevics H, Jansen H, Kremsner PG, Efferth T, Kumar D. A Randomised, Double Blind, Placebo-Controlled Pilot Study of Oral Artesunate Therapy for Colorectal Cancer. EBioMedicine. 2014 Nov 15;2(1):82-90. doi: 10.1016/j.ebiom.2014.11.010. PMID: 26137537; PMCID: PMC4484515.
Von Hagens, 2017	Standard Chemothe rapy for breast cancer	Breast cancer	Phase 1	23	100 mg	Daily for four weeks	oral	Not measured	No complete or partial remission reached	Blood and lymphatics: leukopenia (3), neutropenia (3), lymphopenia (3), anemia (3), decrease of reticulocytes	von Hagens C, Walter-Sack I, Goeckenjan M, Osburg J, Storch-Hagenlocher B, Sertel S, Elsässer M, Remppis BA, Edler L, Munzinger J, Efferth T, Schneeweiss A, Strowitzki T. Prospective open

					150 mg<br><br>200 mg				Stable disease in 10/23<br><br>8 patients not eligable for response assessment	Cardiac: NTproBNP increase<br>Muscolosceletal: asthenia (3)<br>Gastrointestinal and nervous adverse events	uncontrolled phase I study to define a well-tolerated dose of oral artesunate as add-on therapy in patients with metastatic breast cancer (ARTIC M33/2). Breast Cancer Res Treat. 2017 Jul;164(2):359-369. doi: 10.1007/s10549-017-4261-1. Epub 2017 Apr 24. PMID: 28439738.
Von Hagens, 2019	Standard chemo-therapy for breast cancer	Breast cancer	Phase 1	13	100 mg<br><br>150 mg<br><br>200 mg	Daily for up to 37 months	oral		Stable disease reched in 6/13	Blood and lymphatics: neutropenia (3), anemia (3), thrombosis (3)<br>Cardiac: arrhythmia<br>Gastrointestinal: diarrhea (3)	von Hagens C, Walter-Sack I, Goeckenjan M, Storch-Hagenlocher B, Sertel S, Elsässer M, Remppis BA, Munzinger J, Edler L, Efferth T, Schneeweiss A, Strowitzki T. Long-term add-on therapy (compassionate use) with oral artesunate in patients with metastatic breast cancer after participating in a phase I study (ARTIC M33/2). Phytomedicine. 2019 Feb 15;54:140-148. doi: 10.1016/j.phymed.2018.09.178. Epub 2018 Sep 17. PMID: 30668363.
Deeken, 2018	None	Advanced solid malignancies	Phase 1	19	8 mg/kg	Average of 2.6 21-day cycles	intravenous	0.015 µg/ml ≈ 0.039 µM	Disease control rate of 27%	Blood and Lymphatics: Anemia (1-4), Neutropenia (1-4), Neutropenic Fever (3/4)<br>Gastrointestinal: Bloating (1/2), Constipation (1-4), Diarrhea (1/2), Reflux (1/2),	Deeken JF, Wang H, Hartley M, Cheema AK, Smaglo B, Hwang JJ, He AR, Weiner LM, Marshall JL, Giaccone G, Liu S, Luecht J, Spiegel JY, Pishvaian MJ. A phase I study of intravenous artesunate in patients with advanced solid tumor malignancies. Cancer Chemother Pharmacol. 2018

					12 mg/kg<br>18 mg/kg<br>25 mg/kg			0.027 µg/ml ≈ 0.07 µM<br>0.068 µg/ml ≈ 0.18 µM<br>0.227 µg/ml ≈ 0.59 µM	No partial or complete response	Nausea (1/2), Vomiting (1-4) Hepatobiliac: ALT/AST increase (1-4), ALP increase (1-4), Bilirubin increase (1-4), Hypalbuminia (1-4) Respiratory: Cough (1/2), Dyspnea (1/2) Muscolosceletal: Asthenia (1/2), Myalgia (1/2), Chestpain (1/2), Backpain (1/2) Nervous System: Headache (1/2), peripheral sensory neuropathy (1/2), Hallucination (3/4), Dizziness (1-4), Dysgeusia (1-4) Dermal: Akne (1/2) Metabolism: Anorexia (1/2), Weightloss (1/2), Hypocalcemia (1/2), Hyponatremia (1/2) General: Chils (1-4), Edema (1-4), Fatigue (1-4)	Mar;81(3):587-596. doi: 10.1007/s00280-018-3533-8. Epub 2018 Feb 1. PMID: 29392450.
Trimble, 2020	None	Cervical intraepithelial neoplasia (CIN 2/3)	Phase 1	28	50 mg	1, 2 or 3 five day cycles	Intravaginal	Not measured	Histologic regression to CIN1 in 19 patients	local symptoms: vaginal pruritus, vaginal pain, vaginal discharge, vaginal spotting,	Trimble CL, Levinson K, Maldonado L, Donovan MJ, Clark KT, Fu J, Shay ME, Sauter ME, Sanders SA, Frantz PS, Plesa M. A first-

					200 mg				Clearance of HPV genotypes in 9/19 patients	vaginal dryness, uterine cramping, pelvic pain, perineal pain, dyspareunia, vaginal yeast infection (2), vaginal inflammation (2), bacterial vaginosis (2), urinary tract infection (2), noninfective cystitis (2) systemic: gastrointestinal discomfort, short-term tinnitus, dizziness, headache considered non-related to study drug: anxiety, insomnia, suicidal ideation, vaginal twitching, fever, flu-like symptoms, body itching, chills, eczema flare	in-human proof-of-concept trial of intravaginal artesunate to treat cervical intraepithelial neoplasia 2/3 (CIN2/3). Gynecol Oncol. 2020 Apr;157(1):188-194. doi: 10.1016/j.ygyno.2019.12.035. Epub 2020 Jan 28. PMID: 32005582.

Table S2 . Peak Plasma Concentrations of Artesunate in different Anti-Malaria trials .

Author, year	*Parasite*	*Study size*	*Dosing*	*Form of application*	*Peak plasma concentration*	*Reference*
Batty, 1998	Plasmodium falciparum	26	120 mg<br><br>100 mg	Intravenous<br><br>Oral	29.5 uM ≈ 11 ug/ml<br><br>2.6 uM ≈ 0.74 ug/ml	Batty KT, Thu LT, Davis TM, Ilett KF, Mai TX, Hung NC, Tien NP, Powell SM, Thien HV, Binh TQ, Kim NV. A pharmacokinetic and pharmacodynamic study of intravenous vs oral artesunate in uncomplicated falciparum malaria. Br J Clin Pharmacol. 1998 Feb;45(2):123-9. doi: 10.1046/j.1365-2125.1998.00655.x. PMID: 9491824; PMCID: PMC1873351.

Batty, 1998	Plasmodium vivax	12	120 mg	Intravenous	13.7 µg/ml ≈ 35,6 µM	Batty KT, Le AT, Ilett KF, Nguyen PT, Powell SM, Nguyen CH, Truong XM, Vuong VC, Huynh VT, Tran QB, Nguyen VM, Davis TM. A pharmacokinetic and pharmacodynamic study of artesunate for vivax malaria. Am J Trop Med Hyg. 1998 Nov;59(5):823-7. doi: 10.4269/ajtmh.1998.59.823. PMID: 9840605.
Ilett, 2002	Plasmodium falciparum	23	120 mg	Intravenous	16.14 µg/ml ≈ 42 µM	Ilett KF, Batty KT, Powell SM, Binh TQ, Thu le TA, Phuong HL, Hung NC, Davis TM. The pharmacokinetic properties of intramuscular artesunate and rectal dihydroartemisinin in uncomplicated falciparum malaria. Br J Clin Pharmacol. 2002 Jan;53(1):23-30. doi: 10.1046/j.0306-5251.2001.01519.x. PMID: 11849191; PMCID: PMC1874553.
Byakika-Kibwika, 2012	Plasmodium falciparum	14	2.4 mg/kg	Intravenous	3.26 µg/ml ≈ 8,48 µM	Byakika-Kibwika P, Lamorde M, Mayito J, Nabukeera L, Mayanja-Kizza H, Katabira E, Hanpithakpong W, Obua C, Pakker N, Lindegardh N, Tarning J, de Vries PJ, Merry C. Pharmacokinetics and pharmacodynamics of intravenous artesunate during severe malaria treatment in Ugandan adults. Malar J. 2012 Apr 27;11:132. doi: 10.1186/1475-2875-11-132. PMID: 22540954; PMCID: PMC3489518.
